# Investigating the Mechanism of *Scutellariae barbata* Herba in the Treatment of Colorectal Cancer by Network Pharmacology and Molecular Docking

**DOI:** 10.1155/2021/3905367

**Published:** 2021-08-02

**Authors:** Xiangjun Qi, Hongbin Xu, Peng Zhang, Guoming Chen, Zhiqiang Chen, Caishan Fang, Lizhu Lin

**Affiliations:** ^1^The First Clinical Medical College of Guangzhou University of Chinese Medicine, Guangzhou 510405, China; ^2^The First Affiliated Hospital of Guangzhou University of Chinese Medicine, Guangzhou 510405, China

## Abstract

**Background:**

Colorectal cancer (CRC) is one of the most common gastrointestinal tumors, which accounts for approximately 10% of all diagnosed cancers and cancer deaths worldwide per year. *Scutellariae barbatae* Herba (SBH) is one of the most frequently used traditional Chinese medicine (TCM) in the treatment of CRC. Although many experiments have been carried out to explain the mechanisms of SBH, the mechanisms of SBH have not been illuminated fully. Thus, we constructed a network pharmacology and molecular docking to investigate the mechanisms of SBH.

**Methods:**

We adopted active constituent prescreening, target predicting, protein-protein interaction (PPI) analysis, Gene Ontology (GO) analysis, Kyoto Encyclopedia of Genes and Genomes (KEGG) analysis, differentially expressed gene analysis, and molecular docking to establish a system pharmacology database of SBH against CRC.

**Results:**

A total of 64 active constituents of SBH were obtained and 377 targets were predicted, and the result indicated that quercetin, luteolin, wogonin, and apigenin were the main active constituents of SBH. Glucocorticoid receptor (NR3C1), pPhosphatidylinositol 4,5-bisphosphate 3-kinase catalytic subunit alpha isoform (PIK3CA), cellular tumor antigen p53 (TP53), transcription factor AP-1 (JUN), mitogen-activated protein kinase 1 (MAPK1), Myc protooncogene protein (MYC), cyclin-dependent kinase 1 (CDK1), and broad substrate specificity ATP-binding cassette transporter ABCG2 (ABCG2) were the major targets of SBH in the treatment of CRC. GO analysis illustrated that the core biological process regulated by SBH was the regulation of the cell cycle. Thirty pathways were presented and 8 pathways related to CRC were involved. Molecular docking presented the binding details of 3 key targets with 6 active constituents.

**Conclusions:**

The mechanisms of SBH against CRC depend on the synergistic effect of multiple active constituents, multiple targets, and multiple pathways.

## 1. Introduction

Colorectal cancer (CRC) is one of the most common gastrointestinal tumors. Nowadays, CRC accounts for approximately 10% of all diagnosed cancers and cancer deaths worldwide per year [1]. The amounts of CRC patients will increase to 2.5 million in 2035 around the world according to the prediction of WHO [1, 2]. Previous studies have indicated that the majority of CRC cells originate from colon stem cells, which were located at the base of colonic crypts [3, 4], and several risk factors such as smoking [5], excessive alcohol ingestion [6], and red meat consumption [7] have been identified to contribute to the genetic mutations of colon stem cells. The therapies for CRC include endoscopic treatment, surgery, radiotherapy, chemotherapy, and immunotherapy [1]. Although there is brilliant progress in diagnostic techniques and treatment strategies [8], there still exist a sizeable percentage of patients at an advanced stage of CRC along with a high degree of metastasis when diagnosed [9]. In addition, the cost of anticancer drugs keeps increasing, which brings heavy economic burden to families, especially in developing countries [10]. Thus, it is necessary to search for more cost-effective and less toxic drugs.

Traditional Chinese medicine (TCM) has a 5000-year history in China, and it is presently regarded as a useful complementary and alternative medication worldwide [11]. Many historic studies have shown that TCM could induce apoptosis, tumor growth, angiogenesis, and metastasis [12–15]. TCM also plays a role in ameliorating the side effects engendered by radiotherapy and chemotherapy [16]. *Scutellariae barbatae* Herba (also known as BanZhiLian in Chinese) is the dried full plant of *Scutellaria barbata* D. Don in the Lamiaceae family, which has been used for thousands of years in China as a “heat-clearing and detoxifying” drug [17, 18]. Moreover, the anticancer effect of SBH has been examined in many types of cancer including lung cancer, hepatic cancer, breast cancer, colorectal cancer, leukemia, and prostate cancer [17]. Although a considerable amount of literature has been published on searching the mechanisms of SBH, the potential mechanisms of SBH have not yet been systematically investigated.

The traditional drug discovery paradigm focuses on screening exquisite ligands, and the presupposition of “one drug for one target for one disease” is accepted by the majority of researchers [19]. However, this paradigm is not suitable for TCM, which is characterized by multiple components, multiple targets, and multiple pathways [20]. Network pharmacology highlights the consolidation of drug targets, biological network, and pharmacology network [19], which provides a feasible method for TCM exploration. It has already been used for searching core active constituents and targets and detecting the mechanisms of TCM.

Molecular docking is an essential tool in computer-assisted drug design, which was firstly proposed in the mid-80s with the purpose of predicting mode of ligand and protein and virtually screening digital compound libraries to reduce expense and speed up drug discovery. The accuracy of molecular docking keeps increasing with the development of computing power and hardware capability.

In this study, we adopted network pharmacology and molecular docking to investigate the mechanisms of SBH in the treatment of CRC. Our work involved 6 parts as follows: (1) collecting active constituents of SBH from online TCM databases and literatures; (2) predicting the targets of SBH and disease targets of CRC; (3) constructing network to show the interaction between SBH and CRC; (4) conducting function analysis including Gene Ontology (GO) analysis and Kyoto Encyclopedia of Genes and Genomes (KEGG) pathway analysis; (5) seeking differentially expressed genes of CRC with a bioinformatics analysis; (6) implementing molecular docking after a literature retrieval.

## 2. Materials and Methods

**Figure 1:**
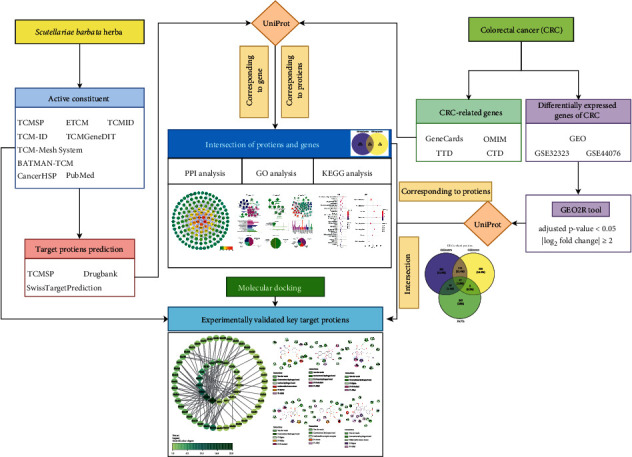
Overall design of the study.

A detailed diagram used to describe the overall design of this study is shown in Figure 1.

### 2.1. Data Sources of Network Pharmacology

#### 2.1.1. Active Constituents of SBH and Target Proteins Prediction

The active constituents of SBH were obtained in 3 steps. Step 1: data retrieval was performed with 7 databases: Traditional Chinese Medicine Systems Pharmacology Database and Analysis Platform (TCMSP, http://lsp.nwu.edu.cn/tcmsp.php) [21], The Encyclopedia of Traditional Chinese Medicine (ETCM, http://www.nrc.ac.cn:9090/ETCM/) [22], Traditional Chinese Medicines Integrated Database (TCMID, http://www.megabionet.org/tcmid/) [23], Traditional Chinese Medicine Information Database (TCM-ID, http://bidd.nus.edu.sg/group/TCMsite/) [24], TCM-Mesh System (http://mesh.tcm.microbioinformatics.org/) [25], TCMGeneDIT Databases (http://tcm.lifescience.ntu.edu.tw/index.html) [26], and Bioinformatics Analysis Tool for Molecular Mechanism of Traditional Chinese Medicine (BATMAN-TCM, http://bionet.ncpsb.org/batman-tcm/) [27]. The benefit of this approach is that all the databases provide a comprehensive study for Chinese herb. The oral bioavailability (OB) and drug-likeness (DL) were adopted to find out the active constituents with better pharmacokinetics [28], and active constituents with OB ≥ 30% and DL ≥ 0.18 were selected for subsequent network construction [29]. Step 2: Anticancer Herbs Database of Systems Pharmacology (CancerHSP, http://lsp.nwsuaf.edu.cn/CancerHSP.php) [30] was retrieved to collect the active constituents with anticancer activity, which contains 2439 anticancer herbal medicines with 3575 anticancer active constituents. In addition, it also provides 832 targets of the active constituents that are predicted by state-of-art methods or collected from the literature. Step 3: to obtain the active constituents which showed bioavailability activities for CRC but were not recorded in aforementioned databases, we constructed a text mining in PubMed using “Scutellariae Barbata Herba” and “cancer” as search terms. After manual filtering of the search results, barbatin F, barbatin G [31], barbatin H [32], SPS2p [33], and SBPW3 [34] were supplemented.

Target protein prediction was based on TCMSP database and DrugBank database (https://www.drugbank.ca/) [35]. Active constituents, whose targets could not be predicted in TCMSP or DrugBank, were predicted by SwissTargetPrediction (http://www.swisstargetprediction.ch), and the top 15 predicted targets were selected for each result of the predicted target classes [36, 37].

To better visualize the relationship between active constituents and target proteins, we coded each active constituent and the SBH active constituent-target protein network was generated using Cytoscape (v3.7.1) software (http://www.cytoscape.org/) [38]. We used Network Analyzer (a cytoscape plugin) to calculate the degree and betweenness centrality of the network. The degree of an active constituent *n* is the number of target proteins linked to *n*. The betweenness centrality reflects the extent to which a node acts as a communication intermediate in the network.

#### 2.1.2. CRC-Related Genes and Corresponding Proteins

CRC-related genes were obtained from 4 databases: GeneCards: the Human Gene Database (https://www.genecards.org/), which integrates more than 190 data source about gene, disease, pathway, and compound [39]; Online Mendelian Inheritance in Man (OMIM, https://omim.org/), which contains information on all known Mendelian disorders and over 15000 genes [40]; Therapeutic Target Database (TTD, http://db.idrblab.net/ttd/), which is a database to provide therapeutic proteins and nucleic acid targets, the targeted disease, and the correspondence between drugs and these targets [41]; Comparative Toxicogenomics Database (CTD, http://ctdbase.org/), which supplies information of 3 interactions: chemical-gene/protein, chemical-disease, and gene-disease [42]. Four databases were searched with the same keywords: “colorectal cancer” or “colorectal Neoplasms” or “Colorectal Carcinoma” or “Colorectal Tumor”, and the species was limited to *Homo sapiens*. The proteins corresponding to the CRC-related genes were standardized using UniProt database for subsequent analysis.

#### 2.1.3. Potential Active Target Proteins (PATPs)

Finally, we took the intersection between the target proteins of SBH and CRC-related proteins as the PATPs for subsequent analysis.

### 2.2. Data Analysis of Network Pharmacology

#### 2.2.1. Protein-Protein Interaction (PPI) Analysis

To figure out the interactions between PATPs, we used the STRING database (https://string-db.org/) to construct the PPI network [43], the species was limited to *Homo sapiens*, and we selected confidence score as 0.95 in the minimum (low confidence < 0.4, medium ≤ 0.7, and high > 0.7). The PPI data was exported as a.tsv file for further analysis. Afterward, we used CytoNCA (a Cytoscape plugin) to evaluate the PPI network [44], and the PPI network was constructed by the top 150 proteins.

#### 2.2.2. Gene Ontology (GO) Analysis

The GO enrichment analysis is one of the most common procedures for determining potential molecular mechanisms of drugs. We did GO biological process (BP) analysis using MCODE (a Cytoscape plugin) [45] and ClueGO (a Cytoscape plugin) [46]. First, the PPI data were filtered in Cytoscape using MCODE plugin and the top 3 clusters (sorted by cluster score) were analyzed by the ClueGo plugin. During this procedure, the significance level of GO terms was set to 0.05, and the species was limited to *Homo sapiens*.

#### 2.2.3. Kyoto Encyclopedia of Genes and Genomes (KEGG) Pathway Analysis

Traditionally, one of the most well-known methods of exploring biological pathways and potential biological functions is KEGG pathway enrichment analysis. In our study, KEGG pathway analysis was performed with *ClusterProfiler* package of R language [47], and the significance level of GO terms was set to 0.05 and species was limited to *Homo sapiens*.

### 2.3. Bioinformatics Analysis

#### 2.3.1. Microarray Data

Microarray data was collected from the GEO database (https://www.ncbi.nlm.nih.gov/geo/) with the following limits: “colorectal cancer” (keyword), “*Homo sapiens*” (study organism), and “tissue” (attribute name). After collection, the GSE32323 dataset, which contained 17 pairs of matched CRC samples based on the GPL570 platform, and the GSE44076 dataset, which contained 98 pairs of matched CRC samples based on the GPL13667 platform, were selected for seeking differentially expressed genes of CRC.

#### 2.3.2. Identification of Differentially Expressed Genes

GEO2R (http://www.ncbi.nlm.nih.gov/geo/geo2r/), an online web tool based on limma package of R language, is one of the most common tools of determining differentially expressed genes (DEGs) of upregulation or downregulation. In our study, we used GEO2R to finish this procedure, and the results were presented as a table of genes. We set adjusted *p* value <0.05 and |log  2 fold change (FC)| ≥ 2 as a criterion to screen out DEGs. The proteins corresponding to the DEGs are standardized using UniProt database for subsequent molecular docking.

### 2.4. Molecular Docking between Key Targets and Active Constituents

#### 2.4.1. Determination of Key Target Proteins and Active Constituents

We took the intersection between PATPs and DEGs-related proteins as the key target proteins for molecular docking. Afterward, we plotted a network diagram to represent the correspondence between key target proteins and active constituents of SBH. Finally, a literature review was conducted to learn the relationship between key target proteins and CRC, and those that were experimentally validated as potential therapeutic targets for CRC would be selected for molecular docking.

#### 2.4.2. Molecular Docking Simulation

First, a suitable protein structure of each target was obtained from the RCSB Protein Data Bank (https://www.rcsb.org/) [48], and the suitable protein was required to satisfy the following 3 conditions as far as possible: (1) the protein owned a 3D structure with a high resolution; (2) the protein owned one or more original ligands; (3) the ligand owned a similar structure with active constituent. Second, Chimera (v1.14) software was applied to remove the heteroatom and water molecule from the proteins and divide the proteins into ligands and receptors. The ligand and receptor files were converted into a pdbqt file then by AutoDockTools (v1.5.6) software. Third, the 2D structures of active constituents were downloaded from the PubChem website (https://pubchem.ncbi.nlm.nih.gov/) [49]. AutoDockTools (v1.5.6) software was used to convert them into a pdbqt file then. Fourth, a grid box size set as 40 × 40 × 40 points with a Vina spacing of 1.0 Å was generated. Finally, we used AutoDock Vina (v1.1.2), an open-source program for molecular docking simulation, to accomplish the docking stimulation, which significantly improved the average accuracy of the binding mode predictions and speed of docking when compared with AutoDock [50]. The result of docking was analyzed by Discovery Studio.

## 3. Results

### 3.1. SBH Active Constituent-Target Network

In our study, we obtained 64 active constituents in total. All active constituents were ranked and detailed information of each active constituent is shown in Table 1. The result of SBH active constituent-target network is presented in Supplementary Figure S1, which was composed of 441 nodes (including 64 active constituent nodes and 377 target nodes) and 1161 edges, and the result of the top 10 active constituents and targets according to the degree is set out in Supplementary Table S1.

### 3.2. Potential Active Target Proteins and PPI Network

**Figure 2:**
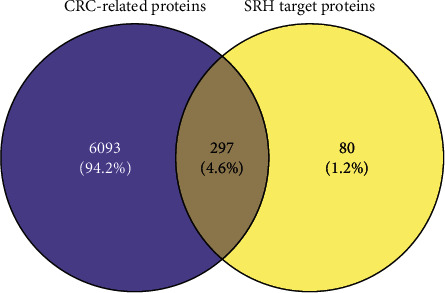
Potential active target proteins.

**Figure 3:**
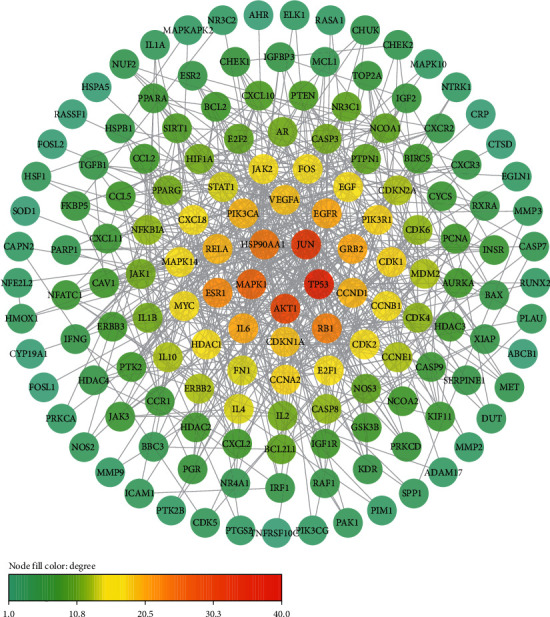
PPI network of potential active target proteins. The nodes represent proteins, and the color of the node represents the degree value.

A total of 377 targets of SBH and 6390 target genes of CRC were obtained, and the intersection of the two groups was regarded as PATPs (Figure 2). A total of 297 proteins were identified and used for constructing the PPI network. After limiting the species to *Homo sapiens* and setting a minimum confidence score as 0.95, 150 proteins were retained as shown in Figure 3. TP53, JUN, AKT1, MAPK1, PIK3CA, etc. were the core proteins of PPI.

### 3.3. GO Enrichment Analysis

**Figure 4:**
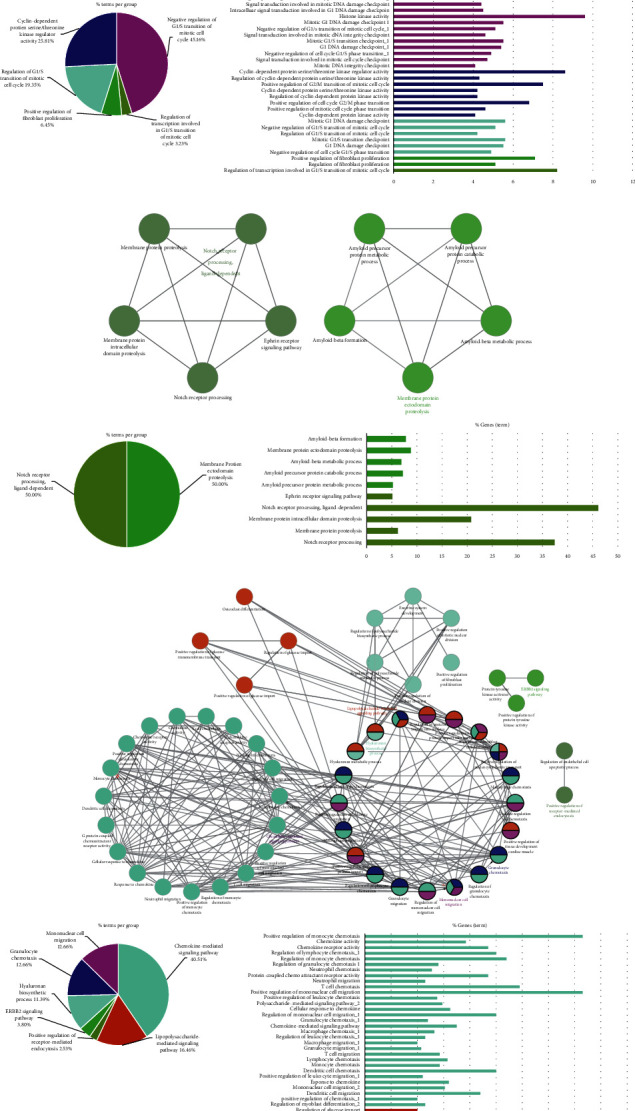
GO enrichment analysis of clusters 1–3 (a–c). For the whole figure, different colors represent different biological process groups. The network diagrams represent the connections between biological process groups, the pie charts represent the proportion of each group, and the bar charts represent the detailed term of each group.

We constructed a cluster analysis using MCODE before GO enrichment analysis, and 9 clusters were obtained altogether (Table 2). Afterwards, the top 3 clusters with high cluster scores were used for the procedure of GO enrichment analysis as shown in Figure 4. Interestingly, the majority of biological processes in cluster 1 were described as follows: negative regulation of G1/S transition of mitotic cell cycle (45.16%), cyclin-dependent protein serine/threonine kinase regulator activity (25.81%), and regulation of G1/S transition of mitotic cell cycle (19.35%), and they are of great importance to cell proliferation and influence the occurrence of cancer. Cluster 2 was constructed by membrane protein ectodomain proteolysis and notch receptor processing, which is ligand-dependent. Cluster 3 was composed of chemokine-mediated signaling pathway (40.51%), lipopolysaccharide-mediated signaling pathway (16.46%), mononuclear cell migration (12.66%), etc.

### 3.4. KEGG Pathway Analysis

**Figure 5:**
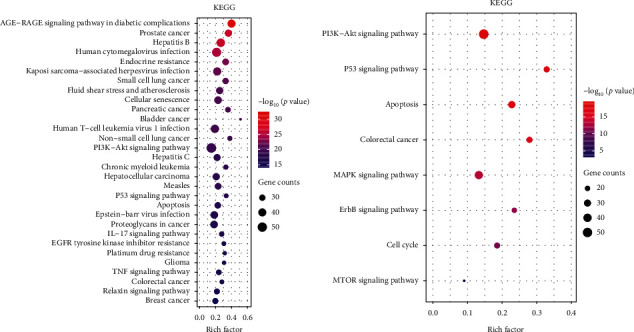
KEGG pathway analysis of potential active targets. (a) For the top 30 terms sorted by adjusted *p* value and (b) for the eight pathways related to CRC summarized by KEGG website. The color of each term represents the different adjusted *p* values <  0.05 and the size of each term represents the gene counts.

KEGG pathway analysis was conducted with the *ClusterProfiler* package of *R* language, and 174 significant pathways (adjusted *p* value < 0.05) were identified. We sorted them with adjusted *p* value and the top 30 terms are shown in Figure 5(a). Eight KEGG pathways related to human solid cancers including prostate cancer, small cell lung cancer, pancreatic cancer, bladder cancer, non-small cell lung cancer, hepatocellular carcinoma, colorectal cancer, and breast cancer were gathered. KEGG website summarized 9 pathways associated with CRC, eight of which were significantly enriched in our results (Figure 5(b)). Notably, the result of the colorectal cancer pathway is significant with adjusted *p* value < 0.05.

### 3.5. Identification of Key Target Protein and Active Constituent

**Figure 6:**
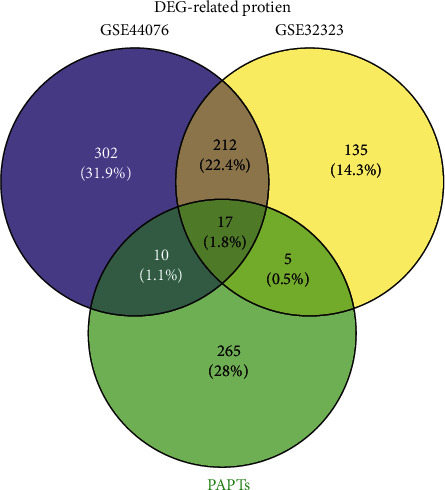
Intersections of differentially expressed genes related proteins and potential active target proteins.

**Figure 7:**
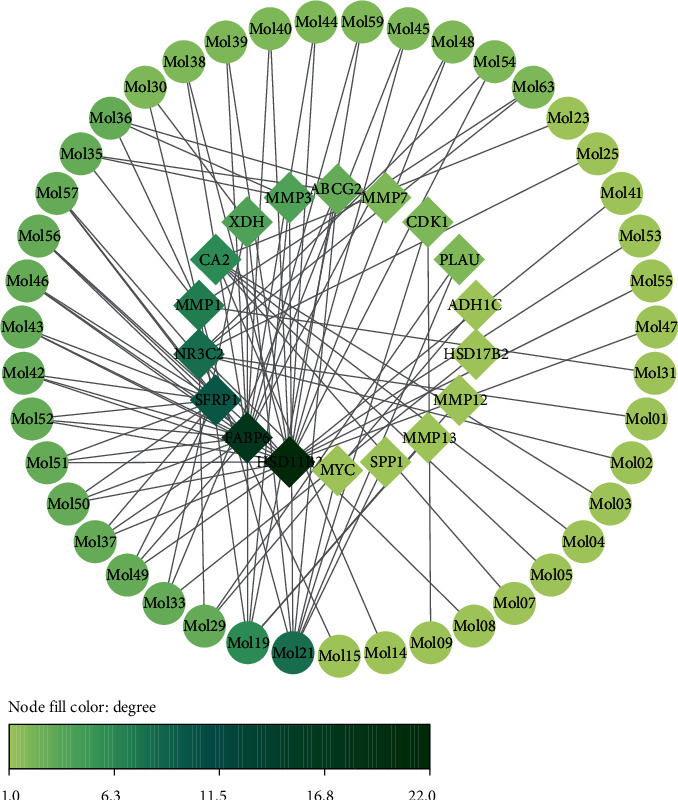
The network of key target proteins and related active constituents. The circular nodes represent the key target proteins, and the diamond nodes represent the active constituents. The color of the node represents the degree value.

In this section, we did an analysis of DEGs in CRC firstly using GEO2R. Two datasets including GSE32323 and GSE44076 were screened from the GEO database. A total of 369 DEGs were obtained from the GSE32323 dataset and 541 DEGs were obtained from the GSE44076. We normalized the proteins corresponding to these DEGs by the STRING database. Seventeen key target proteins were remaining after intersecting with PATPs (Figure 6): ABCG2, SFRP1, MMP7, MYC, CA2, NR3C2, HSD17B2, PLAU, ADH1C, HSD11B2, MMP3, XDH, FABP6, CDK1, MMP1, MMP12, and SPP1. We constructed a network to show the active constituents that corresponded to these target proteins as presented in Figure 7. Among the active constituents mapped by key target proteins, quercetin, luteolin, baicalein, etc., were identified to be the top 10 active constituents in terms of degree value in the SBH active constituent-target network. After a literature review of these key target proteins, we chose MYC, ABCG2, and CDK1 for molecular docking.

### 3.6. Molecular Docking Analysis

**Figure 8:**
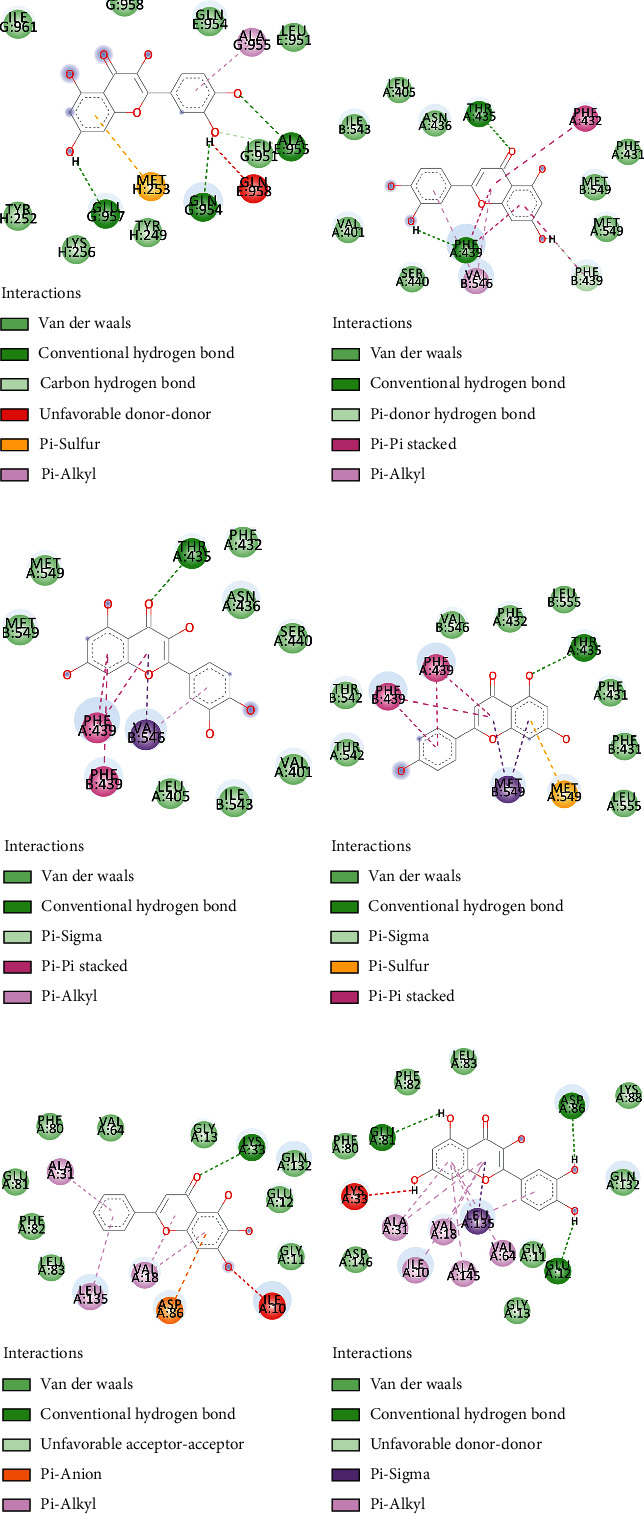
Molecular docking of key target proteins and active constituents. (a) For quercetin and MYC. (b–d) For luteolin, apigenin, and quercetin, respectively. (e) For baicalein and CDK1. (f) For quercetin and CDK1.

To verify how an active constituent binds to target as previously referred to a molecular docking using Autodock Vina was developed in this section. We predicted whether an active constituent could enter the active pocket of the target protein successfully and calculated the affinities between them. We summarized the affinities of the selected active constituents with interactive residues and the count of hydrogen bonds formed between interactive residues, which are set out in Table 3 and Figure 8.

#### 3.6.1. Docking of Quercetin on MYC

As shown in Table 3 and Figure 8, the binding affinity of this combination was −7.6 kcal/mol, and GLN958, GLN954, ALA955, MET253, and GLU957 were identified as interactive residues. Quercetin was bound with MYC by forming 4 hydrogen bonds with GLN954, GLN957, and ALA955. The MET253 residue was found to form a pi-sulfur and the ALA955 residue was formed with pi-alkyl. In addition, there were 8 van der Waals interactions between quercetin and ILE961, LYS256, GLU957, TYR252, TYR259, LEU951, GLN954, and GLN958.

#### 3.6.2. Docking of the 3 Active Constituents on ABCG2

As presented in Table 3, the binding affinity of the luteolin upon ABCG2 was −8 kcal/mol. The PHE439 residue interacted with luteolin by forming one hydrogen bond and the THR435 residue formed one hydrogen bond. The binding affinity between apigenin and ABCG2 was the highest among 3 integrations. Although only one hydrogen bond was found between apigenin and ABCG2, we observed 8 van der Waals interactions between apigenin and ILE543, MET549, VAL401, PHE432, LEU405, ASN436, and SER440. Interestingly, the PHE439 residue formed 3 pi-pi stacked interactions. As for quercetin, one hydrogen bond, four pi-alkyl interactions, and one pi-anion interactions were identified.

#### 3.6.3. Docking of the 2 Active Constituents on CDK1

The binding affinity of 2 active constituents on CDK1 was −8 kcal/mol. From Figure 8, there were 3 hydrogen bonds provided by the GLU81, ASP86, and GLU12 residues in the interaction with quercetin. In addition, the PHE80, PHE82, LEU83, GLY11, GLY13, and ASP146 residues constituted 6 van der Waals interactions. However, there was no hydrogen bond between baicalein and CDK1.

## 4. Discussion

SBH has been used in TCM for thousands of years, the function of which is described as “heat-clearing and detoxifying” in TCM therapeutic principle and the *Chinese Pharmacopoeia 2015*. In spite of that, SBH has been used in various cancers, specially treated with CRC [17].

In our study, we investigated the potential mechanisms of SBH on CRC and predicted the pivotal active constituents and target proteins. The SBH active constituent-target network consisted of 64 active constituents and 441 targets, revealing the pharmacological foundation of SBH. The potential active constituents and targets were deduced in this section. The majority of potential active constituents of SBH are flavonoids. Quercetin (degree = 147) is a polyphenolic flavonoid with underlying anticancer activity, which exists ubiquitously in the vegetal food source, especially in various traditional Chinese medicine [51]. Accumulation of in vitro and in vivo studies has concentrated on potential chemopreventive activity and underlying mechanisms of quercetin in CRC. In vitro studies have identified the following effects of quercetin: induction of cell cycle arrest at the G1 phase, induction of apoptosis and autophagy, decreased expression of cyclooxygenase-2 (COX-2), and heat shock protein synthesis (HSP90AA1, degree = 18), etc. [52–57]. In vivo studies have found that quercetin regulated proliferation and apoptosis via suppressed expression of cyclooxygenase-1 (COX-1), COX-2, and inducible nitric oxide synthase (iNOS) [58]. Luteolin (degree = 93) is a naturally occurring flavonoid, which also serves as a dietary flavonoid [59]. The effects of luteolin in CRC have been proved similar to quercetin such as cell growth inhibition and induction of apoptosis [60, 61]. Wogonin (degree = 55) and baicalein (degree = 37) also belong to the flavonoid, and both of them have shown a significant antitumor effect that has been verified experimentally on CRC cells [62]. In addition, apigenin (degree = 40), beta-sitosterol (degree = 37), stigmasterol (degree = 31), etc., which are not flavonoids but are confirmed to have effects on CRC in vivo or in vitro, also contribute to the primary potential active constituents. A total of 441 targets were predicted and we noticed that many targets were contacted with several active constituents such as NR3C1, HSD11B2, PTGS2, CYP23B1, and PTGS1, and we speculated the top 10 targets could be important in the treatment of CRC as shown in Supplementary Table S1. NR3C1, a receptor of glucocorticoids [63], plays an important role in inflammatory responses and cellular proliferation and differentiation [64]. However, NR3C1 (degree = 24) has been identified likely to be a CRC suppressor gene [65]. Moreover, NR3C1 was proved as a differentially expressed gene in CRC by bioinformatics analysis [66]. Prostaglandin G/H synthase 2 (PTGS2, degree = 21) and prostaglandin G/H synthase 1 (PTGS1, degree = 19) are crucial enzymes in the conversion of arachidonate to prostaglandin H2 (PGH2), which are well known as the targets of nonsteroidal anti-inflammatory drugs (NSAIDs) such as aspirin and ibuprofen [67]. Previous research has established that inhibition of the PGHSs with NSAIDs reduced the development of colon cancer [68]. PTGS2-positive patients were faced with an increased risk of CRC recurrence and poorer CRC-specific survival [69]. In particular, PIK3CA, the gene coding for PI3K p110*α* and the catalytic subunit of PI3K [70], was found in this network. The mutation of PIK3CA exists in approximately 15–20% of CRC [71], which influences the activation of PI3K-AKT signaling pathway and mTOR signaling pathway [72–74]. PI3KCA was simultaneously targeted by 2 active constituents: scutebarbatine E and scutebarbatine N. Scutebarbatine E is a kind of *neo*-clerodane diterpenoid alkaloid extracted from SBH, and an in vitro experiment has shown significant cytotoxic activities against HT29 CRC cells [75]. Scutebarbatine N belongs to norditerpenoid alkaloids, which also showed significant cytotoxic activities in HT29 CRC cells [76]. However, their specific mechanisms have not been reported yet, so our study contributes to this aspect.

We defined the intersection of SBH target proteins and CRC-related proteins as the PATPs. In order to describe the target protein's function fully, a PPI network was constructed using the PATPs, as could be seen in Figure 3. TP53, JUN, AKT1, MAPK1, etc. were the core proteins of PPI. To date, previous studies have revealed that TP53 is a tumor suppressor gene in many tumor types, which induces cell cycle arrest and apoptosis [77]. TP53 mutation is the most typical phenomenon in human cancers [78]. An international collaborative study has reported the occurrence of TP53 mutations in CRC, which was found in 34% of the proximal colon tumors as well as 45% of the distal colon and rectal tumors [79]. Although the mechanisms of TP53 mutation are not understood fully, there is no doubt TP53 has a great capability as a therapeutic strategy in the future. JUN was identified to be bound to the USP28 (a nuclear-localized deubiquitinase related to DNA damage response checkpoint and MYC protooncogene stability) promoter and involved in GTPase KRas (KRAS)-mediated transcriptional activation of USP28 in CRC [80]. AKT1 is one of the AKT kinases, involved in many biological processes such as proliferation, cell survival growth, and angiogenesis, and the therapeutic potential of inhibitors targeting PI3K-AKT pathway in cancer has been discussed [81]. PI3K-AKT pathway plays a pivotal role in the mechanisms of traditional Chinese medicine, which are used frequently when treated with CRC. The majority of them are involved in the inhibition of PI3K-AKT pathway through SRC and AKT1 [82]. Three MAP kinases (MAPK) including MAPK1, MAPK14, and MAPK10 are found in Figure 3, and MAPK plays a crucial role in the MAPK/ERK cascade [83]. Historically, research has investigated that MAPK1 and MAPK14 were associated with cancer risk and survival in CRC [84]. In addition to the 4 proteins mentioned above, other proteins, such as VEGFA, IL6, CDK1, MYC, could also induce the survival of CRC cells. Therefore, we speculated that one of the core functions of the PPI network was involved in the regulation of cancer cells.

KEGG pathway analysis was accomplished using *R* package *ClusterProfiler*, and the most significant 30 of 174 pathways are set out in Figure 5. The most striking result emerging from the data is that colorectal cancer pathway (adjusted *p* value: 4.89*E* − 15) is significant, and the results also indicate that SBH has the potential to treat diverse cancers such as prostate cancer [85, 86], lung cancer [87, 88], breast cancer [86, 89, 90], and pancreatic cancer [91], which has been confirmed by previous studies. The KEGG website has summarized 9 pathways related to colorectal cancer and we investigated the enrichment information in our study of these pathways. Eight of 9 pathways were found in our study including MAPK signaling pathway (adjusted *p* value: 1.46*E* − 12), ErbB signaling pathway (adjusted *p* value: 2.82*E* − 11), cell cycle pathway (adjusted *p* value: 1.18*E* − 10), p53 signaling pathway (adjusted *p* value: 7.10*E* − 17), mTOR signaling pathway (adjusted *p* value: 0.001478074), PI3K-Akt signaling pathway (adjusted *p* value: 1.58*E* − 18), apoptosis pathway (adjusted *p* value: 1.78*E* − 16), and Wnt signaling pathway (adjusted *p* value: 7.62*E* − 05). It indicates that the therapeutical effects of TCM depend on the cooperation of multiple pathways. We speculated the PI3K-AKT signaling pathway was the most important pathway, which was the most significant among them with a total of 52 genes gathered. With many fundamental cellular functions such as proliferation, growth, and survival identified [92, 93], the potential of PI3K-AKT signaling pathway in cancer has already been discussed [81, 94]. Inhibition of PI3K has become a new therapeutic strategy in CRC with PIK3CA mutation [95]. Interestingly, PI3K-AKT signaling pathway corresponds to mTOR signaling pathway. mTOR, a serine/threonine protein kinase, is known as an important downstream effector of AKT and related to the activation of AKT [96]. In addition, MAPK signaling pathway is interconnected with PI3K-AKT signaling pathway [97], and approximately 30–40% CRC patients harbor a mutation in KRAS [70], which is a part of RAS-RAF-MAPK cascade joining in the cellular function of CRC cells [98]. Therefore, inhibition of both MAPK and PI3K-AKT signaling pathway could be a more effective strategy. Recurrent genetic alterations of Wnt signaling pathway occur in the majority of CRC. The adenomatous polyposis coli (APC) is a tumor suppressor gene and regarded as a central hub in early CRC, and mutations in the APC often dysregulate the Wnt signaling pathway [99]. Our findings indicate that SBH produces the healing efficacy for CRC possibly by regulating the pathways mentioned above.

Three key target proteins were selected for molecular docking. MYC is a protooncogene encoding nuclear transcription factor and regulates tumorigenesis [100], which also serves as a downstream effector of Wnt and Ras signaling pathways in CRC [101]. Genetic ablation of c-Myc, a member of the MYC family, suppresses intestinal tumorigenesis in CRC mouse models [102]. Moreover, the inhibitor of c-Myc was identified to be advantageous in anticancer, such as 10058-F4, which could induce apoptosis and differentiation of acute myeloid leukemia cell [103]. Thus, MYC plays an important role in further CRC therapy [104], and in vitro experiment has proved that quercetin induced apoptosis in HT-29 cells and reduced expression of c-Myc [54]. Our molecular docking results indicated that quercetin has a good docking affinity with MYC. The previous set of analyses has identified that quercetin was a pivotal active constituent of SBH, which formed 3 hydrogen bonds with CDK1, and that the binding energy was −8 kcal/mol. Though there is no hydrogen bond found between baicalein and CDK1, baicalein shared the same affinity with quercetin. CDK1 is a serine/threonine kinase which belongs to the CDKs family and regulates the cell cycle, and it has been wildly accepted that CDK1 is the only essential cell cycle CDK [105]. The nonselective inhibitor of CDK-dinaciclib has been shown to arrest cell-cycle progression and inhibit tumor growth [106]. In recent years, literature has identified that CDK1 is a mediator of apoptosis resistance in CRC [107]. Among the three active constituents including quercetin, luteolin, and apigenin targeted ABCG2, apigenin seems to be the most promising active constituent. Multidrug resistance (MDR) is a pivotal factor influencing the efficacy of chemotherapy and the prognosis of tumor patients. Overexpression of adenosine triphosphate (ATP)-binding cassette (ABC) transporter family is one of the most important mechanisms of MDR, and the major ABC transporters include P-glycoprotein (P-gp/ABCB1), breast cancer resistance protein (BCRP/ABCG2), and multidrug resistance-associated protein 2(MRP2/ABCC2) [108]. Importantly, one of the mechanisms of resistance to irinotecan in CRC is that ABCG2 gene encodes ABC efflux transporter and reduces intracellular drug accumulation [109], so targeting ABCG2 is an effective therapeutic principle to enhance the efficacy of irinotecan [110]. Interestingly, it has been reported that decreasing expression of ABCG2 and ABCB5 may induce the depletion of c-Myc and enhance the chemosensitivity of colon cancer stem cells (CSCs) [111]. We did not perform molecular docking analyses for the remaining 14 of the 17 key targets, due to the lack of sufficiently strong evidence for their use as therapeutic targets at this time. The expression level of HSD11B2 gene was significantly increased in CRC tissues, and the ectopic expression of HSD11B2 gene promoted the metastasis of CRC [112]. FABP6, hypermethylated SFRP1, XDH, PLAU, ADH1C, HSD17B2, and SPP1 have been identified more as a colorectal cancer biomarker than as a therapeutic target at present [113–119]. NR3C2, CA2, and MMP1 were identified as key target proteins in another network pharmacological pharmacology analysis of the colorectal cancer. Jin et al. [120] performed molecular docking between these proteins and quercetin, stigmasterol, and baicalein which were also identified in our study as shown in Figure 7. MMP1, MMP3, MMP7, MMP12, and MMP13 belong to the matrix metalloproteinase family, and MMPs were able to participate in the tumor metastasis process by degrading the ECM. However, MMP2 and MMP9 were significantly related to colorectal cancer and could be regulated by Chinese medicine [121]. These targets are closely related to CRC, which may become potentially therapeutic targets and provide reference for future research.

## 5. Conclusions

Traditional Chinese medicine is characterized by multiple components, multiple targets, and multiple pathways. Network pharmacology, along with molecular docking, bioinformation, and system biology, provides an available methodology to uncover the complex therapeutical effects of TCM. Prior studies have confirmed that SBH presents noticeable antitumor effects. We set out to investigate the potential mechanisms of SBH and hunt pivotal active constituents, targets, and pathways.

In this study, a total of 64 active constituents of SBH were obtained from 7 TCM databases and literature, and these active constituents were associated with 377 targets, which were mapped to predicted targets of CRC to get 297 common targets treated as PATPs. After that, a PPI network was constructed to demonstrate the interactions between PATPs. The second major investigation was that we did a GO and KEGG analysis using PATPs. Finally, we did a differentially expressed gene analysis of CRC and 229 DEGs were obtained. After the DEGs related proteins were mapped to PATPs, seventeen key target proteins were remaining for molecular docking. The result indicated that quercetin, luteolin, wogonin, and apigenin were the effective active constituents of SBH. NR3C1, PIK3CA, TP53, JUN, MAPK1, MYC, CDK1, and ABCG2 were the major targets of SBH in the treatment of CRC. The most obvious finding emerging from GO analysis was that the core biological process regulated by SBH was the regulation of cell cycle. One of the most significant findings from KEGG analysis was that pathways were significantly enriched in CRC and its related pathways. Molecular docking results reveal that SBH's active constituents have an acceptable binding affinity with MYC, CDK1, and ABCG2, all of which have shown the potential to treat with CRC. Interestingly, we found that targeting MYC and ABCG2 could contribute to enhancing the efficacy of chemotherapy. A limitation of this study is that further experiments are necessary to demonstrate our findings.

## Figures and Tables

**Table 1:** Active constituents of *Scutellariae barbata* herba.

Ranking	Active constituents
Mol01	(2R)-5,7-Dihydroxy-2-(4-hydroxyphenyl)chroman-4-one
Mol02	24-Ethylcholest-4-en-3-one
Mol03	5,7,4′-Trihydroxy-6-methoxyflavanone
Mol04	5,7,4′-Trihydroxy-8-methoxyflavanone
Mol05	5-Hydroxy-7,8-dimethoxy-2-(4-methoxyphenyl)chromone
Mol06	6-Hydroxynaringenin
Mol07	7-Hydroxy-5,8-dimethoxy-2-phenyl-chromone
Mol08	9,19-Cyclolanost-24-en-3-ol
Mol09	Baicalein
Mol10	Baicalin
Mol11	Beta-sitosterol
Mol12	Campesterol
Mol13	Carthamidin
Mol14	Chrysin-5-methylether
Mol15	CLR
Mol16	Daucostero_qt
Mol17	Dinatin
Mol18	Eriodictyol
Mol19	Luteolin
Mol20	Moslosooflavone
Mol21	Quercetin
Mol22	Rhamnazin
Mol23	Rivularin
Mol24	Salvigenin
Mol25	Sitosterol
Mol26	Sitosterol acetate
Mol27	Stigmasta-5,22-dien-3-ol-acetate
Mol28	Stigmastan-3,5,22-triene
Mol29	Stigmasterol
Mol30	Wogonin
Mol31	Scutebarbatine F
Mol32	6-O-Nicotinoylscutebarbatine G
Mol33	Apigenin
Mol34	Scutelinquanine D
Mol35	Scutebarbatine E
Mol36	Scutebarbatine N
Mol37	6-O-(2-Carbonyl-3-methylbutanoyl)scutehenanine A
Mol38	6-O-Acetylscutehenanine A
Mol39	7-O-Nicotinoylscutebarbatine H
Mol40	Barbatellarine B
Mol41	Barbatin C
Mol42	Barbatin E
Mol43	Scutebarbatine C
Mol44	Scutebarbatine D
Mol45	Scutebarbatine H
Mol46	Scutebata A
Mol47	Scutehenanine A
Mol48	Scutehenanine D
Mol49	6,7-Di-O-nicotinoylscutebarbatine G
Mol50	6-O-Nicotinoyl-7-O-acetylscutebarbatine G
Mol51	Barbatin A
Mol52	Barbatin B
Mol53	Barbatin D
Mol54	Pheophorbide a
Mol55	Scutebarbatine B
Mol56	Scutehenanine B
Mol57	Scutehenanine C
Mol58	Scutebarbatine O
Mol59	Scutebarbatine G
Mol60	Scutellarin
Mol61	7-Acetoxybarbatin C
Mol62	6-Acetoxybarbatin C
Mol63	Barbatin F
Mol64	Barbatin H

**Table 2:** Detailed information of 9 clusters using MCODE.

Cluster	Score	Nodes	Edges
1	7.846	14	51
2	5.6	6	14
3	4.211	20	40
4	3.4	11	17
5	3.333	4	5
6	3.333	4	5
7	3	3	3
8	2.933	16	22
9	2.889	10	13

**Table 3:** Molecular interactions of key target and active constituent.

Integration	Targets	Affinity (kcal/mol)	Number of hydrogen bonds	H bond interacting residues
Quercetin	MYC	−7.6	3	GLU957, GLN954, ALA955
Luteolin	ABCG2	−8	2	PHE439, THR435
Quercetin	ABCG2	−7.8	1	THR435
Apigenin	ABCG2	−8.3	1	THR435
Baicalein	CDK1	−8	1	LYS33
Quercetin	CDK1	−8	3	GLU81, ASP86, GLU12

## Data Availability

The data used to support the findings of this study are included within the article.

## Supplementary Materials

Supplementary Materials The result of SBH active constituent-target network along with the top 10 active constituents and targets according to the degree can be found in the supplementary materials.

## References

[1] Bray F., Ferlay J., Soerjomataram I., Siegel R. L., Torre L. A., Jemal A. (2018). Global cancer statistics 2018: GLOBOCAN estimates of incidence and mortality worldwide for 36 cancers in 185 countries. *CA: A Cancer Journal for Clinicians*.

[2] Ferlay J., Ervik M., Lam F., Colombet M., Mery L., Piñeros M. (2018). *Cancer Today (Powered by GLOBOCAN 2018): IARC Cancer Base No. 15*.

[3] Medema J. P. (2013). Cancer stem cells: the challenges ahead. *Nature Cell Biology*.

[4] Nassar D., Blanpain C. (2016). Cancer stem cells: basic concepts and therapeutic implications. *Annual Review of Pathology*.

[5] Raimondi S., Botteri E., Iodice S., Lowenfels A. B., Maisonneuve P. (2009). Gene-smoking interaction on colorectal adenoma and cancer risk: review and meta-analysis. *Mutation Research*.

[6] Cai S., Li Y., Ding Y., Chen K., Jin M. (2020). Alcohol drinking and the risk of colorectal cancer death: a meta-analysis. *European Cancer Prevention Organisation*.

[7] Chan D. S., Lau R., Aune D. (2011). Red and processed meat and colorectal cancer incidence: meta-analysis of prospective studies. *PLoS One*.

[8] Siegel R. L., Miller K. D., Fedewa S. A. (2017). Colorectal cancer statistics. *CA: A Cancer Journal for Clinicians*.

[9] Linnekamp J. F., Wang X., Medema J. P., Vermeulen L. (2015). Colorectal cancer heterogeneity and targeted therapy: a case for molecular disease subtypes. *Cancer Research*.

[10] Prasad V., De Jesus K., Mailankody S. (2017). The high price of anticancer drugs: origins, implications, barriers, solutions. *Nature Reviews. Clinical Oncology*.

[11] Goss P. E., Strasser-Weippl K., Lee-Bychkovsky B. L. (2014). Challenges to effective cancer control in China, India, and Russia. *Lancet Oncology*.

[12] Hsu S.-C., Ou C.-C., Li J.-W. (2008). Ganoderma tsugae extracts inhibit colorectal cancer cell growth via G2/M cell cycle arrest. *Journal of Ethnopharmacology*.

[13] Pan T. L., Hung Y. C., Wang P. W. (2009). Functional proteomic and structural insights into molecular targets related to the growth inhibitory effect of tanshinone IIA on HeLa cells. *Proteomics*.

[14] Pang X., Yi Z., Zhang J. (2010). Celastrol suppresses angiogenesis-mediated tumor growth through inhibition of AKT/mammalian target of rapamycin pathway. *Cancer Research*.

[15] Gao J. L., Shi J. M., He K. (2008). Yanhusuo extract inhibits metastasis of breast cancer cells by modulating mitogen-activated protein kinase signaling pathways. *Oncology Reports*.

[16] Qi F., Li A., Inagaki Y. (2010). Chinese herbal medicines as adjuvant treatment during chemoor radio-therapy for cancer. *Bioscience Trends*.

[17] Wang L., Chen W., Li M., Zhang F., Chen K., Chen W. (2019). A review of the ethnopharmacology, phytochemistry, pharmacology, and quality control of Scutellaria barbata D. Don. *Journal of Ethnopharmacology.*.

[18] Dai Z. J., Lu W. F., Gao J. (2013). Anti-angiogenic effect of the total flavonoids in Scutellaria barbata D. Don. *BMC Complementary and Alternative Medicine*.

[19] Hopkins A. L. (2007). Network pharmacology. *Nature Biotechnology*.

[20] Song Y., Wang H., Pan Y., Liu T. (2019). Investigating the multi-target pharmacological mechanism of hedyotis diffusa willd acting on prostate cancer: a network pharmacology approach. *Biomolecules*.

[21] Ru J., Li P., Wang J. (2014). TCMSP: a database of systems pharmacology for drug discovery from herbal medicines. *Journal of Cheminformatics*.

[22] Xu H. Y., Zhang Y. Q., Liu Z. M. (2019). ETCM: an encyclopaedia of traditional Chinese medicine. *Nucleic Acids Research*.

[23] Huang L., Xie D., Yu Y. (2018). TCMID 2.0: a comprehensive resource for TCM. *Nucleic Acids Research*.

[24] Chen X., Zhou H., Liu Y. B. (2006). Database of traditional Chinese medicine and its application to studies of mechanism and to prescription validation. *British Journal of Pharmacology*.

[25] Zhang R. Z., Yu S. J., Bai H., Ning K. (2017). TCM-Mesh: the database and analytical system for network pharmacology analysis for TCM preparations. *Scientific Reports*.

[26] Fang Y. C., Huang H. C., Chen H. H., Juan H. F. (2008). TCMGeneDIT: a database for associated traditional Chinese medicine, gene and disease information using text mining. *BMC Complementary and Alternative Medicine*.

[27] Liu Z., Guo F., Wang Y. (2016). BATMAN-TCM: a bioinformatics analysis tool for molecular mechanism of traditional Chinese medicine. *Scientific Reports*.

[28] Tao W., Xu X., Wang X. (2012). Network pharmacology-based prediction of the active ingredients and potential targets of Chinese herbal Radix Curcumae formula for application to cardiovascular disease. *Journal of Ethnopharmacology*.

[29] Gong B., Kao Y., Zhang C., Sun F., Zhao H. (2018). Systematic investigation of Scutellariae barbatae Herba for treating hepatocellular carcinoma based on network pharmacology. *Evidence-Based Complementary and Alternative Medicine*.

[30] Tao W., Li B., Gao S. (2015). CancerHSP: anticancer herbs database of systems pharmacology. *Scientific Reports*.

[31] Wang M., Chen Y., Hu P., Ji J., Li X., Chen J. (2018). Neoclerodane diterpenoids from *Scutellaria barbata* with cytotoxic activities. *Natural Product Research*.

[32] Wang M., Ma C., Chen Y., Li X., Chen J. (2018). Cytotoxic neo-clerodane diterpenoids from *Scutellaria barbata* D.don. *Chemistry and Biodiversity*.

[33] Sun P., Sun D., Wang X. (2017). Effects of *Scutellaria barbata* polysaccharide on the proliferation, apoptosis and EMT of human colon cancer HT29 Cells. *Carbohydrate polymers*.

[34] Li H., Su J., Jiang J. (2019). Characterization of polysaccharide from Scutellaria barbata and its antagonistic effect on the migration and invasion of HT-29 colorectal cancer cells induced by TGF-*β*1. *International Journal of Biological Macromolecules*.

[35] Law V., Knox C., Djoumbou Y. (2014). DrugBank 4.0: shedding new light on drug metabolism. *Nucleic Acids Research*.

[36] Daina A., Michielin O., Zoete V. (2019). SwissTargetPrediction: updated data and new features for efficient prediction of protein targets of small molecules. *Nucleic Acids Research*.

[37] Gfeller D., Grosdidier A., Wirth M., Daina A., Michielin O., Zoete V. (2014). SwissTargetPrediction: a web server for target prediction of bioactive small molecules. *Nucleic Acids Research*.

[38] Shannon P., Markiel A., Ozier O. (2003). Cytoscape: a software environment for integrated models of biomolecular interaction networks. *Genome Research*.

[39] Stelzer G., Rosen N., Plaschkes I. (2016). The GeneCards suite: from gene data mining to disease genome sequence analyses. *Current Protocols in Bioinformatics*.

[40] Amberger J. S., Bocchini C. A., Schiettecatte F., Scott A. F., Hamosh A. (2015). OMIM.org: online Mendelian Inheritance in Man (OMIM(R)), an online catalog of human genes and genetic disorders. *Nucleic Acids Research*.

[41] Wang Y., Zhang S., Li F. (2020). Therapeutic target database 2020: enriched resource for facilitating research and early development of targeted therapeutics. *Nucleic Acids Research*.

[42] Davis A. P., Grondin C. J., Johnson R. J. (2017). The comparative toxicogenomics database: update 2017. *Nucleic Acids Research*.

[43] Szklarczyk D., Morris J. H., Cook H. (2017). The STRING database in 2017: quality-controlled protein-protein association networks, made broadly accessible. *Nucleic Acids Research*.

[44] Tang Y., Li M., Wang J., Pan Y., Wu F. X. (2014). CytoNCA: a cytoscape plugin for centrality analysis and evaluation of protein interaction networks. *Biosystems*.

[45] Bandettini W. P., Kellman P., Mancini C. (2012). MultiContrast delayed enhancement (MCODE) improves detection of subendocardial myocardial infarction by late gadolinium enhancement cardiovascular magnetic resonance: a clinical validation study. *Journal of Cardiovascular Magnetic Resonance*.

[46] Bindea G., Mlecnik B., Hackl H. (2009). ClueGO: a Cytoscape plug-in to decipher functionally grouped gene ontology and pathway annotation networks. *Bioinformatics*.

[47] Yu G., Wang L. G., Han Y., He Q. Y. (2011). clusterProfiler: an R package for comparing biological themes among gene clusters. *OMICS: A Journal of Integrative Biology*.

[48] Bernstein F. C., Koetzle T. F., Williams G. J. (1977). The Protein Data Bank: a computer-based archival file for macromolecular structures. *Journal of Molecular Biology*.

[49] Kim S., Chen J., Cheng T. (2019). PubChem 2019 update: improved access to chemical data. *Nucleic Acids Research*.

[50] Trott O., Olson A. J. (2010). AutoDock Vina: improving the speed and accuracy of docking with a new scoring function, efficient optimization, and multithreading. *Journal of Computational Chemistry*.

[51] Gbylik-Sikorska M., Gajda A., Burmanczuk A., Grabowski T., Posyniak A. (2019). Development of a UHPLC-MS/MS method for the determination of quercetin in milk and its application to a pharmacokinetic study. *Journal of Veterinary Research*.

[52] Koishi M., Hosokawa N., Sato M. (1992). Quercetin, an inhibitor of heat shock protein synthesis, inhibits the acquisition of thermotolerance in a human colon carcinoma cell line. *Japanese Journal of Cancer Research*.

[53] Shan B. E., Wang M. X., Li R. Q. (2009). Quercetin inhibit human SW480 colon cancer growth in association with inhibition of cyclin D1 and survivin expression through Wnt/beta-catenin signaling pathway. *Cancer Investigation*.

[54] Yang L., Liu Y., Wang M. (2016). Quercetin-induced apoptosis of HT-29 colon cancer cells via inhibition of the Akt-CSN6-Myc signaling axis. *Molecular Medicine Reports*.

[55] Zhang X. A., Zhang S., Yin Q., Zhang J. (2015). Quercetin induces human colon cancer cells apoptosis by inhibiting the nuclear factor-kappa B Pathway. *Pharmacognosy Magazine*.

[56] Zhao Y., Fan D., Zheng Z. P. (2016). 8-C-(E-phenylethenyl)quercetin from onion/beef soup induces autophagic cell death in colon cancer cells through ERK activation. *Molecular Nutrition & Food Research*.

[57] Darband S. G., Kaviani M., Yousefi B. (2018). Quercetin: a functional dietary flavonoid with potential chemo-preventive properties in colorectal cancer. *Journal of Cellular Physiology*.

[58] Warren C. A., Paulhill K. J., Davidson L. A. (2009). Quercetin may suppress rat aberrant crypt foci formation by suppressing inflammatory mediators that influence proliferation and apoptosis. *The Journal of Nutrition*.

[59] Seelinger G., Merfort I., Wolfle U., Schempp C. M. (2008). Anti-carcinogenic effects of the flavonoid luteolin. *Molecules*.

[60] Pandurangan A. K., Dharmalingam P., Sadagopan S. K., Ramar M., Munusamy A., Ganapasam S. (2013). Luteolin induces growth arrest in colon cancer cells through involvement of Wnt/beta-catenin/GSK-3beta signaling. *Journal of Environmental Pathology, Toxicology and Oncology: Official Organ of the International Society for Environmental Toxicology and Cancer*.

[61] Krifa M., Leloup L., Ghedira K., Mousli M., Chekir-Ghedira L. (2014). Luteolin induces apoptosis in BE colorectal cancer cells by downregulating calpain, UHRF1, and DNMT1 expressions. *Nutrition and Cancer*.

[62] Kim S. J., Kim H. J., Kim H. R. (2012). Antitumor actions of baicalein and wogonin in HT-29 human colorectal cancer cells. *Molecular Medicine Reports*.

[63] Vitellius G., Fagart J., Delemer B. (2016). Three novel heterozygous point mutations of NR3C1 causing glucocorticoid resistance. *Human Mutation*.

[64] Lu N. Z., Cidlowski J. A. (2005). Translational regulatory mechanisms generate N-terminal glucocorticoid receptor isoforms with unique transcriptional target genes. *Molecular Cell*.

[65] Matthews L. C., Berry A. A., Morgan D. J. (2015). Glucocorticoid receptor regulates accurate chromosome segregation and is associated with malignancy. *Proceedings of the National Academy of Sciences of the United States of America*.

[66] Wu S., Wu F., Jiang Z. (2017). Identification of hub genes, key miRNAs and potential molecular mechanisms of colorectal cancer. *Oncology Reports*.

[67] Lucido M. J., Orlando B. J., Vecchio A. J., Malkowski M. G. (2016). Crystal structure of aspirin-acetylated human cyclooxygenase-2: insight into the formation of products with reversed stereochemistry. *Biochemistry*.

[68] Smith W. L., DeWitt D. L., Garavito R. M. (2006). Cyclooxygenases: structural, cellular, and molecular biology. *Annual Review of Biochemistry*.

[69] Kunzmann A. T., Murray L. J., Cardwell C. R., McShane C. M., McMenamin U. C., Cantwell M. M. (2013). PTGS2 (Cyclooxygenase-2) expression and survival among colorectal cancer patients: a systematic review. *Cancer Epidemiology, Biomarkers & Prevention*.

[70] De Roock W., De Vriendt V., Normanno N., Ciardiello F., Tejpar S. (2011). KRAS, BRAF, PIK3CA, and PTEN mutations: implications for targeted therapies in metastatic colorectal cancer. *Lancet Oncology*.

[71] Rowan A. J., Lamlum H., Ilyas M. (2000). APC mutations in sporadic colorectal tumors: a mutational “hotspot” and interdependence of the “two hits. *Proceedings of the National Academy of Sciences of the United States of America*.

[72] Samuels Y., Velculescu V. E. (2004). Oncogenic mutations of PIK3CA in human cancers. *Cell Cycle*.

[73] Kang S., Bader A. G., Vogt P. K. (2005). Phosphatidylinositol 3-kinase mutations identified in human cancer are oncogenic. *Proceedings of the National Academy of Sciences of the United States of America*.

[74] Engelmann J. C., Rahmann S., Wolf M. (2008). Modelling cross-hybridization on phylogenetic DNA microarrays increases the detection power of closely related species. *Molecular Ecology Resources*.

[75] Dai S. J., Chen M., Liu K., Jiang Y. T., Shen L. (2006). Four new neo-clerodane diterpenoid alkaloids from Scutellaria barbata with cytotoxic activities. *Chemical & Pharmaceutical Bulletin (Tokyo)*.

[76] Dai S. J., Peng W. B., Shen L., Zhang D. W., Ren Y. (2011). New norditerpenoid alkaloids from *Scutellaria barbata* with cytotoxic activities. *Natural Product Research*.

[77] Li X. L., Zhou J., Chen Z. R., Chng W. J. (2015). P53 mutations in colorectal cancer—molecular pathogenesis and pharmacological reactivation. *World Journal of Gastroenterology*.

[78] Kandoth C., McLellan M. D., Vandin F. (2013). Mutational landscape and significance across 12 major cancer types. *Nature*.

[79] Russo A., Bazan V., Iacopetta B. (2005). The TP53 colorectal cancer international collaborative study on the prognostic and predictive significance of p53 mutation: influence of tumor site, type of mutation, and adjuvant treatment. *Journal of Clinical Oncology: Official Journal of the American Society of Clinical Oncology*.

[80] Serra R. W., Fang M., Park S. M., Hutchinson L., Green M. R. (2014). A KRAS-directed transcriptional silencing pathway that mediates the CpG island methylator phenotype. *Elife*.

[81] Engelman J. A. (2009). Targeting PI3K signalling in cancer: opportunities, challenges and limitations. *Nature Reviews Cancer*.

[82] Zhu H., Hao J., Niu Y., Liu D., Chen D., Wu X. (2018). Molecular targets of Chinese herbs: a clinical study of metastatic colorectal cancer based on network pharmacology. *Scientific Reports*.

[83] Qi M., Elion E. A. (2005). MAP kinase pathways. *Journal of Cell Science*.

[84] Slattery M. L., Lundgreen A., Wolff R. K. (2012). MAP kinase genes and colon and rectal cancer. *Carcinogenesis*.

[85] Wong B. Y., Nguyen D. L., Lin T. (2009). Chinese medicinal herb Scutellaria barbata modulates apoptosis and cell survival in murine and human prostate cancer cells and tumor development in TRAMP mice. *European Journal of Cancer Prevention: The Official Journal of the European Cancer Prevention Organisation (ECP)*.

[86] Marconett C. N., Morgenstern T. J., San Roman A. K., Sundar S. N., Singhal A. K., Firestone G. L. (2010). BZL101, a phytochemical extract from the Scutellaria barbata plant, disrupts proliferation of human breast and prostate cancer cells through distinct mechanisms dependent on the cancer cell phenotype. *Cancer Biology & Therapy*.

[87] Chen C. C., Kao C. P., Chiu M. M., Wang S. H. (2017). The anti-cancer effects and mechanisms of *Scutellaria barbata* D. Don on CL1-5 lung cancer cells. *Oncotarget*.

[88] Gong T., Wang C. F., Yuan J. R. (2015). Inhibition of tumor growth and immunomodulatory effects of flavonoids and scutebarbatines of *Scutellaria barbata* D. Don in lewis-bearing C57bl/6 mice. *Evidence-Based Complementary and Alternative Medicine*.

[89] Zheng X., Kang W., Liu H., Guo S. (2018). Inhibition effects of total flavonoids from Sculellaria barbata D. Don on human breast carcinoma bone metastasis via downregulating PTHrP pathway. *International Journal of Molecular Medicine*.

[90] Fong S., Shoemaker M., Cadaoas J. (2008). Molecular mechanisms underlying selective cytotoxic activity of BZL101, an extract of Scutellaria barbata, towards breast cancer cells. *Cancer Biology & Therapy*.

[91] Song L., Chen X., Wang P., Gao S., Qu C., Liu L. (2018). Effects of baicalein on pancreatic cancer stem cells via modulation of sonic Hedgehog pathway. *Acta Biochimica et Biophysica Sinica*.

[92] Engelman J. A., Luo J., Cantley L. C. (2006). The evolution of phosphatidylinositol 3-kinases as regulators of growth and metabolism. *Nature Reviews. Genetics*.

[93] Song G., Ouyang G., Bao S. (2005). The activation of Akt/PKB signaling pathway and cell survival. *Journal of Cellular and Molecular Medicine*.

[94] Dasari A., Messersmith W. A. (2010). New strategies in colorectal cancer: biomarkers of response to epidermal growth factor receptor monoclonal antibodies and potential therapeutic targets in phosphoinositide 3-kinase and mitogen-activated protein kinase pathways. *Clinical Cancer Research: An Official Journal of the American Association for Cancer Research*.

[95] Fernandes M. S., Sanches J. M., Seruca R. (2019). Targeting the PI3K signalling as a therapeutic strategy in colorectal cancer. *Advances in Experimental Medicine and Biology*.

[96] Saxton R. A., Sabatini D. M. (2017). mTOR signaling in growth, metabolism, and disease. *Cell*.

[97] Britten C. D. (2013). PI3K and MEK inhibitor combinations: examining the evidence in selected tumor types. *Cancer Chemotherapy and Pharmacology*.

[98] Pylayeva-Gupta Y., Grabocka E., Bar-Sagi D. (2011). RAS oncogenes: weaving a tumorigenic web. *Nature Reviews Cancer*.

[99] Morin P. J., Sparks A. B., Korinek V. (1997). Activation of beta-catenin-Tcf signaling in colon cancer by mutations in beta-catenin or APC. *Science*.

[100] Dang C. V. (2013). MYC, metabolism, cell growth, and tumorigenesis. *Cold Spring Harbor Perspectives in Medicine*.

[101] Cancer N., Atlas G. (2012). Comprehensive molecular characterization of human colon and rectal cancer. *Nature*.

[102] Sansom O. J., Meniel V. S., Muncan V. (2007). Myc deletion rescues Apc deficiency in the small intestine. *Nature*.

[103] Huang M. J., Cheng Y. C., Liu C. R., Lin S., Liu H. E. (2006). A small-molecule c-Myc inhibitor, 10058-F4, induces cell-cycle arrest, apoptosis, and myeloid differentiation of human acute myeloid leukemia. *Experimental Hematology*.

[104] Elbadawy M., Usui T., Yamawaki H., Sasaki K. (2019). Emerging roles of C-Myc in cancer stem cell-related signaling and resistance to cancer chemotherapy: a potential therapeutic target against colorectal cancer. *International Journal of Molecular Sciences*.

[105] Santamaria D., Barriere C., Cerqueira A. (2007). Cdk1 is sufficient to drive the mammalian cell cycle. *Nature*.

[106] Ascierto P. A., McArthur G. A., Dreno B. (2016). Cobimetinib combined with vemurafenib in advanced BRAF(V600)-mutant melanoma (coBRIM): updated efficacy results from a randomised, double-blind, phase 3 trial. *Lancet Oncology*.

[107] Zhang P., Kawakami H., Liu W. (2018). Targeting CDK1 and MEK/ERK overcomes apoptotic resistance in BRAF-mutant human colorectal cancer. *Molecular Cancer Research*.

[108] Chen Z., Shi T., Zhang L. (2015). Mammalian drug efflux transporters of the ATP binding cassette (ABC) family in multidrug resistance: a review of the past decade. *Cancer Letters*.

[109] Bates S. E., Medina-Perez W. Y., Kohlhagen G. (2004). ABCG2 mediates differential resistance to SN-38 (7-ethyl-10-hydroxycamptothecin) and homocamptothecins. *The Journal of Pharmacology and Experimental Therapeutics*.

[110] Nielsen D. L., Palshof J. A., Brunner N., Stenvang J., Viuff B. M. (2017). Implications of ABCG2 expression on irinotecan treatment of colorectal cancer patients: a review. *International Journal of Molecular Sciences*.

[111] Zhang H. L., Wang P., Lu M. Z., Zhang S. D., Zheng L. (2019). c-Myc maintains the self-renewal and chemoresistance properties of colon cancer stem cells. *Oncology Letters*.

[112] Chen J., Liu Q. M., Du P. C. (2020). Type-2 11*β*-hydroxysteroid dehydrogenase promotes the metastasis of colorectal cancer via the Fgfbp1-AKT pathway. *American Journal of Cancer Research*.

[113] Zhang Y., Zhao X., Deng L. (2019). High expression of FABP4 and FABP6 in patients with colorectal cancer. *World Journal of Surgical Oncology*.

[114] Miao Y., Li Q., Wang J. (2020). Prognostic implications of metabolism-associated gene signatures in colorectal cancer. *PeerJ*.

[115] Liu X., Fu J., Bi H. (2019). DNA methylation of SFRP1, SFRP2, and WIF1 and prognosis of postoperative colorectal cancer patients. *BMC Cancer*.

[116] Žlajpah M., Boštjančič E., Zidar N. (2020). (Epi)genetic regulation of osteopontin in colorectal cancerogenesis. *Epigenomics*.

[117] Offermans N. S. M., Ketcham S. M., van den Brandt P. A., Weijenberg M. P., Simons C. (2018). Alcohol intake, ADH1B and ADH1C genotypes, and the risk of colorectal cancer by sex and subsite in The Netherlands Cohort Study. *Carcinogenesis*.

[118] Lin M., Zhang Z., Gao M., Yu H., Sheng H., Huang J. (2019). MicroRNA-193a-3p suppresses the colorectal cancer cell proliferation and progression through downregulating the PLAU expression. *Cancer Management and Research*.

[119] Lin J. H., Manson J. E., Kraft P. (2011). Estrogen and progesterone-related gene variants and colorectal cancer risk in women. *BMC Medical Genetics*.

[120] Jin J., Chen B., Zhan X., Zhou Z., Liu H., Dong Y. (2021). Network pharmacology and molecular docking study on the mechanism of colorectal cancer treatment using Xiao-Chai-Hu-Tang. *PLoS One*.

[121] Yan X., Li M., Chen L. (2020). *α-*Solanine inhibits growth and metastatic potential of human colorectal cancer cells. *Oncology Reports*.

