# Supplementary material for: Investigating the Mechanism of Scutellariae barbata Herba in the Treatment of Colorectal Cancer by Network Pharmacology and Molecular Docking
Source: Evid Based Complement Alternat Med. 2021 Aug 2;2021:3905367. doi: 10.1155/2021/3905367 (PMC8352706; doi:10.1155/2021/3905367)

Supplementary Table S1. Top 10 Active constituents and targets sorted by degree

Active constituent	Degree	BetweennessCentrality	Target	Degree	BetweennessCentralit
quercetin	147	0.4228802	NR3C1	24	0.1294543
luteolin	93	0.2227119	HSD11B2	22	0.0068073
wogonin	55	0.0763245	PTGS2	21	0.0767229
Apigenin	40	0.0648765	CYP27B1	21	0.0045999
beta-sitosterol	37	0.0774998	PTGS1	19	0.0324061
baicalein	37	0.0557213	HSP90AA1	18	0.0264951
Stigmasterol	31	0.0741788	FNTA	18	0.0032711
7-hydroxy-5,8-dimethoxy-2-phenyl-chromone	26	0.0097444	AVPR1B	18	0.0032535
5-hydroxy-7,8-dimethoxy-2-(4-methoxyphenyl)chromone	26	0.0147921	SRD5A1	17	0.0018075
Moslosooflavone	25	0.0088645	FABP6	17	0.0029422

Supplementary Figure S1. SBH active constituent-target network. Hexagonal nodes represent the active constituents of SBH and round nodes represent the related targets

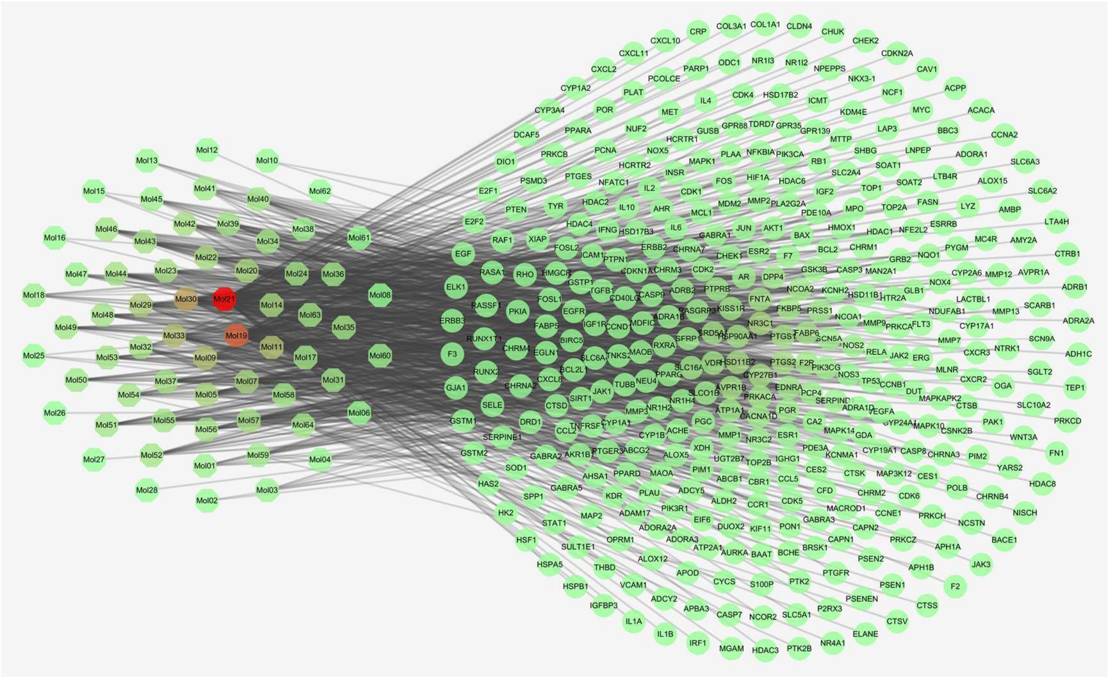

Supplement: Supplementary Materials — The result of SBH active constituent-target network along with the top 10 active constituents and targets according to the degree can be found in the supplementary materials. [file 3905367.f1.pdf]
